# Pancreatectomy and Pancreatic Surgery

**DOI:** 10.3390/life13061400

**Published:** 2023-06-16

**Authors:** Beata Jabłońska, Sławomir Mrowiec

**Affiliations:** Department of Digestive Tract Surgery, Medical University of Silesia, 40-752 Katowice, Poland; mrowasm@poczta.onet.pl

Pancreatectomy, including pancreaticoduodenectomy (PD), as well as central pancreatectomy (CP), distal pancreatectomy (DP) and total/subtotal pancreatectomy, is a major, complex and difficult surgical procedure performed for various benign and malignant pancreatic diseases: from chronic pancreatitis, through benign cystic tumors and neuroendocrine neoplasms to malignant neoplasms, including pancreatic ductal adenocarcinoma (PDAC) [1].

PD is a resection of the pancreatic head and duodenum indicated in pancreatic head cancer, chronic pancreatitis, periampullary tumors, and other lesions of the pancreatic head. [2]. PD is commonly referred to as Whipple surgery after Dr. Allen Whipple, the surgeon who presented the technique in the 1930’s. It is indicated for PDAC and other periampullary cancers, including distal bile duct cholangiocarcinoma, adenocarcinoma of the ampulla of Vater, and duodenal adenocarcinoma. Less common indications for PD include neuroendocrine tumors, gastrointestinal stromal tumors (GISTs), mucinous cystic neoplasms, sarcomas, and isolated metastatic lesions in the pancreatic head. A “classic” PD involves the resection of the distal part of the stomach, including the pylorus, en bloc with the pancreatic head, common bile duct, duodenum, and gallbladder. In the pylorus-preserving PD, the stomach and pylorus are not removed. This PD was first described by Dr. Watson in 1944 and was later popularized by Drs. Traverso and Longmire. The initial goal of this procedure was to reduce postoperative reflux, dumping, diarrhea, and weight loss by preserving the pylorus. Currently, it is already known that Traverso PD is related to a higher risk of delayed gastric emptying (DGE) [3].

An elective total pancreatectomy (TP) was first performed by Eugene Rockey of Portland, Oregon, in 1942 [4]. Indications for TP are the following: malignant tumors of the pancreatic head with involvement in the left pancreas, the inability to obtain tumor-free R0 resections at the pancreatic margin, inability to perform pancreatic anastomosis or a high risk of postoperative pancreatic fistula (POPF) following PD (due to local intraoperative conditions such as atrophic fatty pancreas or due to poor general condition), recurrent pancreatic cancer in the remnant pancreas, the removal of the remaining pancreas after Whipple complication (pancreatic bleeding or leak), multifocal intraductal papillary mucinous neoplasm (IPMN) in all parts of the pancreas, multifocal pancreatic neuroendocrine tumors with a history of multiple endocrine neoplasia, hereditary pancreatic cancer or family history (this indication is controversial), and intractable pain due to chronic pancreatitis or multiple bouts of recurrent acute pancreatitis [1,4].

A special form of TP is TP with autologous islet cell transplantation (TPAIT). It is performed in order to prevent postoperative diabetes and its serious complications following TP. The current indications for this procedure include small-duct painful chronic pancreatitis, hereditary/genetic pancreatitis (HGP), as well as less frequent indications such as benign/borderline pancreatic tumors (IPMNs, neuroendocrine neoplasms) and “high-risk pancreatic stump”. The use of TPAIT in malignant pancreatic and peripancreatic neoplasms has been reported in the worldwide literature, but currently is not a standard but rather a controversial management in these patients [5].

DP is the resection of the pancreatic tail and/or body with or with no concomitant splenectomy. Indications for DP are as follows: benign or malignant tumors involving the pancreatic body or tail (located to the left side of the superior mesenteric vein), chronic pancreatitis within the pancreatic body or tail, a pseudocyst involving the pancreatic tail, trauma of the distal pancreas, ductal disruption or stricture +/− pancreatic fistula in pancreatic body or tail [6]. DP is performed less frequently compared to PD because of the lower incidence of pancreatic lesions within the left pancreas and the later clinical manifestation of diseases located within this part of the pancreas [7]. In 2003, Strasberg described a new DP technique, termed “radical antegrade modular pancreatosplenectomy” (RAMPS), which is oncologically safe with respect to the dissection planes used to achieve negative margins as well as the extent of lymph node dissection to improve long-term results. In this procedure, the posterior plane of dissection continues left from medial, exposing the left renal vein and clearing Gerota’s fascia off the left kidney, or the dissection continues posteriorly to the diaphragm using the retroperitoneal muscles as the posterior border. The benefit of this surgical approach is to ensure a negative deep margin with complete regional lymph-node dissection [8].

CP generally can be performed as an alternative surgical approach to DP in the treatment of benign or low-grade malignant lesions located in the pancreatic neck and body in order to reduce the loss of parenchyma and therefore postoperative endocrine and exocrine pancreatic failure [9,10]. Thus, CP offers improved endocrine and exocrine long-term results at the expense of a higher risk of postpancreatectomy hemorrhage (PPH) and POPF without increased perioperative mortality [10]. Indications for CP are the following: benign or borderline lesions located in the pancreatic neck or proximal body, inability of enucleation of the lesion located within the pancreatic isthmus/proximal body, and trauma-related injury to the isthmus/proximal body [1,11,12]. According to most authors, CP is less preferred over DP due to a higher rate of POPF. Despite equivalent clinically significant morbidities, long-term results are better after CP compared to DP in low-grade pancreatic body tumors [13].

Recently, pancreatectomies with concurrent vascular resections involving the superior mesenteric and portal veins (SMV-PV), celiac axis (CA), superior mesenteric artery (SMA) and common hepatic artery (CHA) have increased. The careful selection of splenic vein (SV) reconstruction is very important to prevent left-sided portal hypertension (LSPH). In DP, CA and CHA resection is largely accepted, while there is debate on the value of SMA and proper HA (PHA) resection and reconstruction [14,15].

Venous resections currently are a standard surgical treatment of PDAC and are recommended for R0 resection due to its similar morbidity and mortality rates compared to standard PD, whereas arterial resections and reconstructions still remain controversial due to significantly increased rates of postoperative morbidity. Currently, there is no significant benefit of arterial resection during pancreatectomy for PDCA. Due to a higher rate of morbidity and mortality and similar survival rate compared with standard pancreatectomy, arterial resection should be performed only in highly selected patients with borderline and locally advanced PDAC. Novel systemic neoadjuvant treatment regimens, such as FOLFIRINOX, are also very important in the treatment of advanced PDAC [15].

Currently, open, laparoscopic and robotic pancreatic surgery is performed. Minimally invasive PDs (MIPDs), which include laparoscopic (LPD) and robotic (RPD) approaches, are increasingly performed in the USA. MIPDs are generally associated with a longer duration of operation compared to open PD (OPD). An increased duration of operation is related to worse outcomes in OPD; however, the effect of duration of operation on MIPD is not well understood. To conclude, a prolonged duration of operation is associated with worse outcomes following open, laparoscopic, and robotic PD. Therefore, surgeons should to optimize the duration of operation, regardless of the approach to PD [16]. Nassour et al. [17] demonstrated a higher mean number of lymph nodes removed and a higher percentage of adequate lymphadenectomy (≥12 lymph nodes) and shorter duration of hospitalization in RPD compared to OPD. The percentage of positive resection margins (R1), as well short-term results (including 30- and 90-day mortality rate and 30-day readmission rates), median overall survival were similar in RPD and OPD [17].

Robotic surgery has become a promising surgical method in minimally invasive pancreatic surgery due to its three-dimensional visualization, tremor filtration, motion scaling, and better ergonomics. Numerous studies have explored the benefits of robotic distal pancreatectomy (RDP) over laparoscopic distal pancreatectomy (LDP) regarding perioperative safety and feasibility, but no consensus has been achieved yet. A recent meta-analysis by Li et al. [18] showed that RDP was related to a greater benefit compared to LDP for higher spleen preservation in benign and low-grade malignant tumors. In addition, RDP was associated with a lower rate of conversion to laparotomy and shorter duration of postoperative hospitalization, but with higher costs. There was no difference between RDP and LDP regarding postoperative complications, except for 30-day mortality which was significantly lower in RDP compared to LDP. Large prospective randomized controlled trials are required to confirm the above-mentioned results [18].

According to the current IG-MIRP (International Evidence- based Guidelines on Minimally Invasive Pancreas Resection) published in 2020, there are insufficient data to recommend the use of minimally invasive MIPD instead of OPD for the treatment of pancreatic-head PDAC. However, both MIPD and OPD are appropriate management options for selected patients with pancreatic head cancer [19,20].

Apart from large pancreatectomies, various pancreatic drainage procedures are performed. Most commonly, these operations are indicated for patients with chronic pancreatitis. The drainage operations include Puestow, Partington–Rochelle, and Duval procedures. The other procedures are resections with extended drainage, including Beger, and Frey operations. Currently, the Partington–Rochelle, Beger, and Frey procedures are the most frequently performed. The Partington–Rochelle procedure involves lateral (longitudinal) pancreaticojejunostomy (anastomosis between the longitudinally incised main pancreatic duct and Roux-Y jejunal loop). The Beger procedure involves the resection of the pancreatic head, preserving the duodenum. The pancreas is transected at a border between the pancreatic head and body, leaving a thin pancreatic disc between the common bile duct and duodenum. The pancreatic body is drained by end-to-end pancreaticojejunostomy and the pancreatic head disc is drained by side-to-side pancreaticojejunostomy using a Roux-Y jejunal loop. Frey procedure involves coring out of the pancreatic head overlying the main and accessory pancreatic ducts and uncinate process, keeping at least 5 mm pancreatic tissue posteriorly and medially along with opening the main duct in the body and tail. The cored head and opened main duct are drained by lateral pancreaticojejunostomy using a Roux-Y jejunal loop. Beger and Frey operations, as duodenum-preserving pancreatic head resections (DPPHRs) with extended pancreatic duct drainage, are recommended for patients with an inflammatory pancreatic head mass [21,22].

Pancreatectomy is associated with a low mortality rate (<5%) but still a relatively high morbidity, with postoperative complications over 20% even in high-volume pancreatic surgical centers. POPF is the most common and important complication following pancreatectomy [1]. The less frequent complications following pancreatectomy include postoperative biliary fistula (POBF), delayed gastric emptying (DGE), postoperative bleeding, and abscess [23].

POPF is one of the most common complications following pancreatectomy. POPF is noted in in about 20% of patients undergoing PD and 26–31% of patients undergoing DP [23]. According to the International Study Group of Pancreatic Fistula (ISGPF), three types of POPFs are distinguished. POPFs of grade A (not relevant clinically biochemic leak) is the most common type. It does not require any special treatment besides continued drainage, because most of these types of POPF heal without other intervention. Types B and C are clinically relevant POPFs. In a POPF of grade B, manifesting as intraabdominal fluid collection with infection signs, but without organ failure, persistence of a drain in situ for >3 weeks or a percutaneous or endoscopic drainage placed into a small fluid collection is needed. A POPF of grade C is the most severe and is related to sepsis and multisystem organ failure as well as hospitalization in an intensive care unit. This POPF requires surgical treatment, such as completion pancreatectomy, in order to save the patient’ life [1,24,25,26,27,28]. The diagnosis of a POPF is based on two signs: an amylase level in drained fluid more than three times the upper limit of the blood amylase level; and an abnormal clinical course [29].

Soft pancreatic texture, pancreatic lipomatosis, and a small pancreatic duct (<3 mm) are known risk factors for POPF [30]. Numerous POPF risk factors have been suggested, such as a soft pancreas, obesity, diabetes mellitus, a lower geriatric nutritional risk index (GNRI), lower albumin concentrations, blood loss, and prolonged duration of operation or radiotherapy. It has been reported that a soft pancreas, higher BMI, blood transfusion, blood loss, and the operative time were major predictors of POPF [29,31]. A meta-analysis by Peng et al. [32] indicated a soft pancreas, higher body mass index (BMI), blood transfusion, blood loss, and the longer duration of operation as major predictors for POPF [32]. It has been reported that worse nutritional status can be related to a higher POPF [33]. Additionally, POPF is frequently associated with significant morbidity and mortality following PD, including intraabdominal infection, intraabdominal hemorrhage, a prolonged duration of hospitalization, indications for reoperation or less invasive (radiological or endoscopic) interventional therapy, and mortality [34].

The incidence of biliary leakage is lower compared to POPF and occurs in 4–12%. The risk factors for POBF include a tiny and thin-walled bile duct (<5 mm), bile infection, and compromised blood supply [30].

A combined POPF/POBF fistula is related to a significantly higher morbidity and mortality compared to isolated POPF following PD. CP and pancreatic ductal adenocarcinoma are related to a lower risk of POPF, as well as POBF. It can be associated with a rather hard pancreatic tissue, as well as enlarged pancreatic and bile duct diameters in patients with the above-mentioned diseases. In contrast, pancreatic metastasis and serous cystic neoplasm (SCN), a neuroendocrine neoplasm, increase the risk of POPF due to a soft pancreas and small pancreatic ducts [30]. Several surgical pancreaticojejunostomy/pancreaticogastrostomy techniques following PD, as well as pancreatic stump creation or pancreatic transection, have been introduced to reduce the risk of POPF [25,34,35,36,37].

Post-pancreatectomy acute pancreatitis (PPAP) is a newly described postoperative complication, defined by elevated serum amylase sustained ≥48 h postoperatively, radiological findings consistent with acute pancreatitis, and associated clinically relevant features. It was defined by the International Study Group for Pancreatic Surgery (ISGPS) [38]. PPAP can trigger further postoperative complications. The diagnosis is based on biochemical, radiological, and clinical criteria, and involves postoperative serum hyperamylasemia (POH) higher than the institutional upper limit for normal, sustained elevated for at least the first 48 h following surgery, as well as radiologic alterations consistent with PPAP, and associated clinically relevant signs [39].

In conclusion, the most important issues regarding pancreatectomy and pancreatic surgery were mentioned above. We invite original research and review papers focusing on various techniques as well as the short- and long-term results of pancreatic resection, including pancreatectomy-combined vascular resection in pancreatectomy for pancreatic cancer. In addition, papers related to various aspects of pancreatic surgery performed in chronic pancreatitis drainage operations (Puestow, Partington–Rochelle, and Duval procedures), resection operations (partial and subtotal or total pancreatectomies), and resections with extended drainage (Beger and Frey procedures) are invited.

## References

[1] Bishop M.A., Simo K. (2023). Pancreatectomy.

[2] Xiang Y., Wu J., Lin C., Yang Y., Zhang D., Xie Y., Yao X., Zhang X. (2019). Pancreatic reconstruction techniques after pancreaticoduodenectomy: A review of the literature. Expert Rev. Gastroenterol. Hepatol..

[3] Giuliano K., Ejaz A., He J. (2017). Technical aspects of pancreaticoduodenectomy and their outcomes. Chin. Clin. Oncol..

[4] Andrén-Sandberg Å., Ansorge C., Yadav T.D. (2016). Are There Indications for Total Pancreatectomy in 2016?. Dig. Surg..

[5] Jabłońska B., Mrowiec S. (2021). Total Pancreatectomy with Autologous Islet Cell Transplantation—The Current Indications. J. Clin. Med..

[6] Olakowski M., Jabłońska B., Braszczok Ł., Lekstan A., Bednarek P., Bratek A., Bocheńska A., Lampe P. (2012). Distal Pancreatectomy—OWN Experience. Pol. Przegl. Chir..

[7] Čečka F., Jon B., Šubrt Z., Ferko A. (2014). Surgical Technique in Distal Pancreatectomy: A Systematic Review of Randomized Trials. BioMed Res. Int..

[8] Kim H.S., Hong T.H., You Y.-K., Park J.S., Yoon D.S. (2021). Radical antegrade modular pancreatosplenectomy (RAMPS) versus conventional distal pancreatectomy for left-sided pancreatic cancer: Findings of a multicenter, retrospective, propensity score matching study. Surg. Today.

[9] Jabłońska B., Żaworonkow D., Dranka-Bojarowska D., Musialski P., Lampe P. (2011). Middle Pancreatectomy—Own Experience. Pol. Przegl. Chir..

[10] Klotz R., Schilling C., Kuner C., Hinz U., Klaiber U., Holze M., Tjaden C., Loos M., Büchler M.W., Hackert T. (2022). Central pancreatectomy prevents postoperative diabetes. J. Hepato-Biliary-Pancreat. Sci..

[11] Celis Zapata J., Berrospi Espinoza F., Ruiz Figueroa E., Payet Meza E., Chavez Passiuri I., Young Tabusso F. (2005). Pancreatectomia central: Indicaciones y resultados peri operatorios de una técnica de conservación de tejido pancreático [Central pancreatectomy. In-dications and perisurgical results of a pancreatic tissue conservation technique]. Rev. Gastroenterol. Peru..

[12] Pausch T.M., Liu X., Dincher J., Contin P., Cui J., Wei J., Heger U., Lang M., Tanaka M., Heap S. (2023). Middle Segment-Preserving Pancreatectomy to Avoid Pancreatic Insufficiency: Individual Patient Data Analysis of All Published Cases from 2003–2021. J. Clin. Med..

[13] Bansal A.K., Nagari B., Nekarakanti P.K., Pakkala A.K., Thumma V.M., Gunturi S.R.V., Pardasani M. (2022). Is central pancreatectomy an effective alternative to distal pancreatectomy for low-grade pancreatic neck and body tumors: A 20-year single-center propensity score-matched case-control study. J. Hepato-Biliary-Pancreat. Sci..

[14] Pedrazzoli S. (2023). Surgical Treatment of Pancreatic Cancer: Currently Debated Topics on Vascular Resection. Cancer Control.

[15] Jabłońska B., Król R., Mrowiec S. (2022). Vascular Resection in Pancreatectomy—Is It Safe and Useful for Patients with Advanced Pancreatic Cancer?. Cancers.

[16] Williams M.D., Bhama A.R., Naffouje S., Kamarajah S.K., Becerra A.Z., Zhang Y., Pappas S.G., Dahdaleh F.S. (2023). Effect of Operative Time on Outcomes of Minimally Invasive Versus Open Pancreatoduodenectomy. J. Gastrointest. Surg..

[17] Nassour I., Winters S.B., Hoehn R., Tohme S., Adam M.A., Bartlett D.L., Lee K.K., Paniccia A., Zureikat A.H. (2020). Long-term oncologic outcomes of robotic and open pancreatectomy in a national cohort of pancreatic adenocarcinoma. J. Surg. Oncol..

[18] Li P., Zhang H., Chen L., Liu T., Dai M. (2023). Robotic versus laparoscopic distal pancreatectomy on perioperative outcomes: A systematic review and meta-analysis. Updat. Surg..

[19] Asbun H.J., Moekotte A.L., Vissers F.L., Kunzler F., Cipriani F., Alseidi A., D’angelica M.I., Balduzzi A., Bassi C., Björnsson B. (2020). The Miami International Evidence-based Guidelines on Minimally Invasive Pancreas Resection. Ann. Surg..

[20] Olakowski M., Jabłońska B., Mrowiec S. (2023). A chronicle of the pancreatoduodenectomy technique development—From the surgeon’s hand to the robotic arm. Acta Chir. Belg..

[21] Jabłońska B. (2013). Is endoscopic therapy the treatment of choice in all patients with chronic pancreatitis?. World J. Gastroenterol..

[22] Kempeneers M., Issa Y., Ali U.A., Baron R., Besselink M., Büchler M., Erkan M., Castillo C.F.-D., Isaji S., Izbicki J. (2019). International consensus guidelines for surgery and the timing of intervention in chronic pancreatitis. Pancreatology.

[23] Donovan E.C., Prakash L.R., Chiang Y.-J., Bruno M.L., Maxwell J.E., Ikoma N., Tzeng C.-W.D., Katz M.H.G., Lee J.E., Kim M.P. (2023). Incidence of Postoperative Complications Following Pancreatectomy for Pancreatic Cystic Lesions or Pancreatic Cancer. J. Gastrointest. Surg..

[24] Malleo G., Pulvirenti A., Marchegiani G., Butturini G., Salvia R., Bassi C. (2014). Diagnosis and management of postoperative pancreatic fistula. Langenbeck’s Arch. Surg..

[25] Tieftrunk E., Demir I.E., Schorn S., Sargut M., Scheufele F., Calavrezos L., Schirren R., Friess H., Ceyhan G.O. (2018). Pancreatic stump closure techniques and pancreatic fistula formation after distal pancreatectomy: Meta-analysis and single-center experience. PLoS ONE.

[26] Jabłońska B., Lampe P., Dziki A., Matyja A., Śledziński Z., Wallner G. (2014). The Association of Polish Surgeons on Pancreatic Fistulas. Pol. Przegl. Chir..

[27] Mech K., Wysocki Ł., Guzel T., Makiewicz M., Nyckowski P., Słodkowski M. (2018). A review of methods for preventing pancreatic fistula after distal pancreatectomy. Pol. Przegl. Chir..

[28] Bassi C., Marchegiani G., Dervenis C., Sarr M., Hilal M.A., Adham M., Allen P., Andersson R., Asbun H.J., Besselink M.G. (2017). The 2016 update of the International Study Group (ISGPS) definition and grading of postoperative pancreatic fistula: 11 Years After. Surgery.

[29] Malgras B., Dokmak S., Aussilhou B., Pocard M., Sauvanet A. (2023). Management of postoperative pancreatic fistula after pancreaticoduodenectomy. J. Visc. Surg..

[30] Aghalarov I., Beyer E., Niescery J., Belyaev O., Uhl W., Herzog T. (2023). Outcome of combined pancreatic and biliary fistulas after pancreatoduodenectomy. HPB.

[31] Funamizu N., Sogabe K., Shine M., Honjo M., Sakamoto A., Nishi Y., Matsui T., Uraoka M., Nagaoka T., Iwata M. (2022). Association between the Preoperative *C*-Reactive Protein-to-Albumin Ratio and the Risk for Postoperative Pancreatic Fistula following Distal Pancreatectomy for Pancreatic Cancer. Nutrients.

[32] Peng Y.-P., Zhu X.-L., Yin L.-D., Zhu Y., Wei J.-S., Wu J.-L., Miao Y. (2017). Risk factors of postoperative pancreatic fistula in patients after distal pancreatectomy: A systematic review and meta-analysis. Sci. Rep..

[33] Jabłońska B., Lampe P., Mrowiec S. (2020). The influence of nutritional status on the incidence of postoperative complications in patients following distal pancreatectomy. Gastroenterol. Rev..

[34] Zhao A., Zhu Q., Qin X., Wang K., Tan K., Liu Z., Song W., Cheng Q., Li X., Chen Z. (2023). A duct-to-mucosa pancreaticojejunostomy for small main pancreatic duct and soft pancreas in minimally invasive pancreaticoduodenectomy. Surg. Endosc..

[35] Hai H., Li Z., Zhang Z., Cheng Y., Liu Z., Gong J., Deng Y. (2022). Duct-to-mucosa versus other types of pancreaticojejunostomy for the prevention of postoperative pancreatic fistula following pancreaticoduodenectomy. Cochrane Database Syst. Rev..

[36] Cheng Y., Briarava M., Lai M., Wang X., Tu B., Cheng N., Gong J., Yuan Y., Pilati P., Mocellin S. (2017). Pancreaticojejunostomy versus pancreaticogastrostomy reconstruction for the prevention of postoperative pancreatic fistula following pancreaticoduodenectomy. Cochrane Database Syst. Rev..

[37] Wu J.-M., Lin Y.-J., Wu C.-H., Kuo T.-C., Tien Y.-W. (2023). Novel Non-duct-to-Mucosa Pancreaticojejunostomy Reconstruction After Pancreaticoduodenectomy: Focus on the Occurrence of Post-pancreatectomy Hemorrhage and Intra-abdominal Abscess. Ann. Surg. Oncol..

[38] Marchegiani G., Barreto S.G., Bannone E., Sarr M., Vollmer C.M., Connor S., Falconi M., Besselink M.G., Salvia R., Wolfgang C.L. (2022). Postpancreatectomy Acute Pancreatitis (PPAP): Definition and Grading From the International Study Group for Pancreatic Surgery (ISGPS). Ann. Surg..

[39] Holmberg M., Schou J., Larsson P., Syed H.R.S., Gilg S., Sparrelid E., Ghorbani P. (2023). Post-Pancreatectomy Acute Pancreatitis—The New Criteria Fail to Recognize Significant Presentations. J. Gastrointest. Surg..

